# Impact of the "Tobacco control law" on exposure to environmental tobacco smoke in Spain

**DOI:** 10.1186/1471-2458-7-224

**Published:** 2007-08-30

**Authors:** Iñaki Galán, Nelva Mata, Carmen Estrada, Lucía Díez-Gañán, Luis Velázquez, Belén Zorrilla, Ana Gandarillas, Honorato Ortiz

**Affiliations:** 1 Department of Epidemiology. Institute of Public Health. Madrid Regional Authority for Health & Consumer Affairs, Spain; 2 Department of Preventive Medicine. Institute of Public Health. Madrid Regional Authority for Health & Consumer Affairs, Spain; 3 Department for Coordination of the Regional Plan of Tobacco Prevention and Control. Institute of Public Health. Madrid Regional Authority for Health & Consumer Affairs, Spain

## Abstract

**Background:**

The initial evaluations of the introduction of legislation that regulates smoking in enclosed public places in European countries, describe an important effect in the control of exposure to environmental tobacco smoke. However, the evidence is still limited. The objective of this study is to estimate the short-term effects of the comprehensive "Tobacco control law" introduced in Spain on January 2006, which includes a total ban of smoking in workplaces and a partial limitation of smoking in bars and restaurants.

**Methods:**

Cross-sectional, population-based study. The self-reported exposure to environmental tobacco smoke at home, at work, in bars and restaurants of the population aged 18 to 64 years in the Madrid Region during a period prior to the law (October and November 2005; n = 1750) was compared to that of the period immediately after the law came into force (January-July 2006; n = 1252). Adjusted odds ratios (OR) were calculated using logistic regression models.

**Results:**

Passive exposure to tobacco smoke at home has hardly changed. However, at indoor workplaces there has been a considerable reduction: after the law came into force the OR for daily exposure > 0–3 hours versus non-exposure was 0.11 (95% CI: 0.07 to 0.17) and for more than 3 hours, 0.12 (95% CI: 0.09 to 0.18). For fairly high exposure in bars and restaurants versus non-exposure, the OR in the former was 0.30 (95% CI: 0.20 to 0.44) and in the latter was 0.24 (95% CI: 0.18 to 0.32); for very high exposure versus non-exposure they were 0.16 (95% CI: 0.10 to 0.24) and 0.11 (95% CI: 0.07 to 0.19), respectively. These results were similar for the smoking and non-smoking populations.

**Conclusion:**

A considerable reduction in exposure to environmental tobacco smoke in the workplace and, to a lesser extent, in bars and restaurants, is related to the implementation of the "Tobacco control law". Although only initial figures, these results already demonstrate the effectiveness of strategies that establish control measures to guarantee smoke-free places.

## Background

In Spain, smoking is the main risk factor for morbidity and mortality, causing around 55,000 deaths annually [1]. The most recent data for prevalence, from the National Health Survey (2003), show that 31% of the adult population smoke regularly [2], a level that positions Spain at around the average for countries within the European Union [3].

Second-hand smoke exposure causes disease and premature death in children and adults who do not smoke, and there is no risk-free level of exposure [4]. Policies that establish smoke-free public places are an effective strategy to reduce passive exposure to tobacco smoke and, in turn, are an important method of control for the reduction of smoking [5,6]. Until January 1^st ^2006, when the so-called "Tobacco control law" – the Law of Health Measures against Smoking and the Regulation of the Sale, Supply, Consumption and Advertising of Tobacco Products [7] – came into force, Spanish legislation was the oldest and most lax in Europe, whereas now it is one of the most advanced. The approval of this regulatory scheme generated considerable debate, but the most controversial aspect refers to the regulation of smoking in enclosed public spaces, especially the total ban on smoking in workplaces, both public and private, except in the open air, and the partial limitation of smoking in bars and restaurants. This partial ban means that for bars and restaurants of less than 100 square metres, the proprietor has to choose between permitting and banning smoking and must indicate the choice made by signs at the entrance to the premises. In bars and restaurants of more than 100 square metres, a smoking area can be provided if considered necessary, as long as it meets the following requirements: its maximum size is 30% of the total area of the premises, it is physically separate from the non-smoking area, and it has an independent ventilation system.

The objective is to estimate the short-term impact of the "Tobacco control law", which could, thereby, orientate future planning.

## Methods

### Study population

Two independent telephone surveys were carried out among the non-institutionalised population aged 18 to 64 years in the Madrid Region. The first survey was carried out during October and November 2005 (n = 1750) and the second one between the last week of January to July 2006 (n = 1252). Telephone registration databases that cover around 90% of all households were used to select the individuals. The telephone interviews were undertaken using a computer assisted telephone interview (CATI) system [8], with the same interviewers for both surveys. The Ethics Committee of the Autonomous Community of Madrid considered that no ethical approval was required for this study.

### Study variables

Levels of passive exposure to environmental tobacco smoke were gathered in a self-reported form, in the following enclosed spaces: at home, at work, in bars and restaurants. The following questions about smoke exposure at home were addressed to households with more than one member: How many people who live with you regularly smoke at home? How long are you in enclosed spaces with tobacco smoke at home? Do you regularly smoke indoors at home? (question to smokers). The following questions about exposure at work were addressed to people who work in indoor places outside the home, such as offices, premises, warehouses: Do you have a colleague who smokes close to you at work, and does the smoke reach your workplace? How long are you exposed to tobacco smoke in indoor workplaces? Do you smoke in indoor places at work? (question to smokers). The following questions about exposure in bars and restaurants were asked to those who had visited such premises during the last month: In general, how would you describe the atmosphere in bars in terms of tobacco smoke? (very high level, fairly high level, low level, none); In general, how would you describe the atmosphere in restaurants in terms of tobacco smoke? (very high level, fairly high level, low level, none).

### Analysis

The indicators of passive exposure to tobacco smoke collected in the two surveys were initially compared by means of bivariant analysis and, subsequently, the adjusted odds ratios were calculated using binary and multinomial logistic regression models, including the following variables: sex, age (9 categories), educational level (4 categories), number of people living in the household (this variable was only introduced in the variables about exposure at home), and tobacco consumption.

The statistical analysis was carried out using Stata version 7.0 (StataCorp, College Station, 2001).

## Results

The response rate, measured as the number of complete interviews divided by the number of complete plus incomplete interviews and those not conducted (including household refusals and non-contacts), was 77% and 66.1% for the first and second surveys, respectively.

Table 1 outlines the population characteristics of the two samples. The age and sex structures are very similar in both, as is smoking prevalence, the proportion of people who work in indoor places outside the home and that of people who have visited restaurants during the last month. There were statistically significant differences, although only small, in educational level, the average number of people living in a household, and of having visited bars during the last month.

**Table 1:** Characteristics of the samples. Population aged 18 to 64 years. Madrid Region, Spain

	Period prior to the law October-November 2005		Period after the law January-July 2006		
	n	%	n	%	*p-value*
Total	1750	100	1252	100	
*Sex*					0.674^a^
Males	846	48.3	615	49.1	
Females	904	51.7	637	50.9	
*Age group*					0.473^a^
18–29	461	26.3	336	26.8	
30–44	707	40.4	479	38.3	
45–64	582	33.3	437	34.9	
*Educational level*					0.001^a^
University	532	30.5	410	32.7	
Secondary, second level	512	29.4	430	34.3	
Secondary, first level	487	27.9	286	22.8	
Primary or below primary	213	12.2	126	10.1	
*Number of people in the household*	1750	3.5 (mean)	1252	3.3 (mean)	0.000^b^
*People who work in indoor places away from home*	1017	58.1	736	58.8	0.713^a^
*People who have visited bars in the last month*	1551	88.6	1055	84.3	0.000^a^
*People who have visited restaurants in the last month*	1321	75.5	906	72.4	0.054^a^
*Tobacco consumption*					0.550^a^
Non-smokers	1195	68.3	842	67.3	
Current smokers	555	31.7	410	32.7	

*p-value*: Comparison period after the law versus period prior to it coming into force. ^a ^Chi-Square test,^b ^t-test

Table 2 shows the changes in the pattern of passive exposure to tobacco smoke in the periods before and after the "Tobacco control law" came into force.

**Table 2:** Comparison of indicators related to exposure to environmental tobacco smoke in indoor places, before and after the "Tobacco control law" came into force. Population aged 18 to 64 years. Madrid Region, Spain

	Period prior to the law October-November 2005 n = 1750^a^			Period after the law January-July 2006 n = 1252^a^			Period after the law versus period prior to the law		
	n	%	95% CI	n	%	95% CI	Odds ratio	95% CI	*p-value*
***Exposure at home***^b^									
Someone regularly smoking at home	579	34.3	32.1 to 36.6	354	30.5	27.9 to 33.2	0.84^e^	0.71 to 1.00	0.044
Time of exposure (daily)									
No or sporadic exposure	1310	77.7	75.7 to 79.7	922	79.8	77.4 to 82.1	1^f^		
> 0–3 hours	235	13.9	12.3 to 15.7	171	14.8	12.8 to 17.0	1,00	0.80 to 1.25	0.998
More than 3 hours	140	8.3	7.0 to 9.7	62	5.4	4.1 to 6.8	0.65	0.47 to 0.90	0.009
Regular smokers who smoke at home	330	61.3	57.1 to 65.5	237	61.6	56.5 to 66.4	1.07^e^	0.81 to 1.42	0.641
***Exposure at indoor workplaces***^c^									
Close exposure (tobacco smoke reaches their workplace)	412	40.5	37.5 to 43.6	66	9.0	7.0 to 11.3	0.14^e^	0.11 to 0.19	< 0.001
Time of exposure (daily)									
No or sporadic exposure	553	54.5	51.4 to 57.6	669	91.1	88.9 to 93.1	1^f^		
> 0–3 hours	193	19.0	16.6 to 21.6	24	3.3	2.1 to 4.8	0.11	0.07 to 0.17	< 0.001
More than 3 hours	269	26.5	23.8 to 29.3	41	5.6	4.0 to 7.5	0.12	0.09 to 0.18	< 0.001
Regular smokers who smoke at work	197	57.9	52.5 to 63.2	27	10.6	7.1 to 15.1	0.08^e^	0.05 to 0.13	< 0.001
***Exposure in bars and restaurants***^d^									
Environmental tobacco smoke in bars									
None	46	3.0	2.2 to 3.9	86	8.1	6.6 to 10.0	1^f^		
Low level	484	31.2	28.9 to 33.6	495	46.9	43.9 to 50.0	0.54	0.37 to 0.80	< 0.001
Fairly high level	640	41.3	38.8 to 43.8	359	34.0	31.2 to 37.0	0.30	0.20 to 0.44	< 0.001
Very high level	381	24.6	22.4 to 26.8	115	10.9	9.1 to 12.9	0.16	0.10 to 0.24	< 0.001
Environmental tobacco smoke in restaurants									
None	157	11.9	10.2 to 13.8	294	32.4	29.4 to 35.6	1^f^		
Low level	798	60.4	57.7 to 63.1	476	52.5	49.2 to 55.8	0.31	0.25 to 0.39	< 0.001
Fairly high level	261	19.8	17.6 to 22.0	114	12.6	10.5 to 14.9	0.24	0.18 to 0.32	< 0.001
Very high level	105	7.9	6.5 to 9.5	22	2.4	1.5 to 3.7	0.11	0.07 to 0.19	< 0.001

^a ^Total sample size. Differs according to variables used

^b ^Households with more than one person

^c ^People who work in indoor places away from home

^d ^People who have visited such premises in the last month

^e ^Binary logistic regression. Odds ratios adjusted by sex, age, number of people in the household (only for exposure at home), consumption of tobacco, and educational level. Reference categories: Nobody regularly smokes at home; Regular smokers who do not smoke at home; No close exposure at workplace; Regular smokers who do not smoke at work

^f ^Multinomial logistic regression. Odds ratios adjusted by the same variables as in the binary logistic regression models. First category = 1 is the reference category

### Exposure at home

A slight reduction in the proportion of households in which any person smoked was seen in the period after the law came into force: 30.5%, as against 34.3% before the law, with an estimated odds ratio (OR) of 0.84 (95% CI: 0.71 to 1.00) (p = 0.044). The proportion of people exposed for 1–3 hours daily to tobacco smoke at home is very similar in both surveys, 13.9% and 14.8%, but there was a decrease in exposure for more than 3 hours: 8.3% and 5.4%, for the periods prior to and after the law respectively, with an estimated OR = 0.65 (95% CI: 0.47 to 0.90) (p = 0.009).

Among the population of smokers, 61.3% smoked at home in the period before the law, and in the period after the law this figure has remained stable at 61.6%.

### Exposure in indoor workplaces

Prior to the law coming into force, 40.5% of people working in indoor places outside the home stated that they were exposed to tobacco smoke in the workplace, this figure was reduced to 9.0% in the period after the law, with an estimated OR = 0.14 (95% CI: 0.11 to 0.19) (p < 0.001). Moreover, daily exposure time has declined since the law: the OR for daily exposure for 1–3 hours as against non-exposure, for the period after the law as compared to the period preceding the law, was 0.11 (95% CI: 0.07 to 0.17); and 0.12 (95% CI: 0.09 to 0.18) for daily exposure for more than 3 hours as against non-exposure (p < 0.001).

For smokers, 57.9% smoked at work prior to the law, whereas after the law the figure was reduced to 10.6%, with an estimated OR of 0.08 (95% CI: 0.05 to 0.13) (p < 0.001).

### Exposure in bars and restaurants

The proportion of people stating that tobacco smoke did not affect the atmosphere, among those who visited bars during the last month, rose from 3.0% to 8.1% between the period before the law and the period after it came into force. In addition, the perception of having been exposed to an atmosphere with a very high level of tobacco smoke fell from 24.6% to 10.9%. Comparing exposure in atmospheres of low, fairly high and very high levels of tobacco smoke as against non-exposure, for the period after the law as against that which preceded it, the OR were 0.54 (95% CI: 0.37 to 0.80), 0.30 (95% CI: 0.20 to 0.44) and 0.16 (95% CI: 0.10 to 0.24), respectively.

After the law came into force, among people visiting restaurants during the last month the perception that the atmosphere in these establishments was not affected by tobacco smoke increased: from 11.9% to 32.4%, while the perception of a very high level of tobacco smoke fell from 7.9% to 2.4%. Comparing exposure in atmospheres of low, fairly high and very high levels of tobacco smoke as against non-exposure, for the period after the law as against that which preceded it, the OR were 0.31 (95% CI: 0.25 to 0.39), 0.24 (95% CI: 0.18 to 0.32) and 0.11 (95% CI: 0.07 to 0.19), respectively.

Finally, Table 3 shows how the differences in exposure at home, at work, and in bars and restaurants since the law came into force, described for the general population, are similar when the analysis is stratified for the smoking and non-smoking populations.

**Table 3:** Comparison of indicators related to exposure to environmental tobacco smoke in indoor places, before and after the "Tobacco control law" came into force, by tobacco consumption. Population aged 18 to 64 years. Madrid Region, Spain

	Period after the law versus period prior to the law in non-smokers			Period after the law versus period prior to the law in current smokers		
	Odds ratio	95% CI	*p-value*	Odds ratio	95% CI	*p-value*
***Exposure at home***^a^						
Someone regularly smoking at home	0.86^d^	0.69 to 1.08	0.171	0.78^d^	0.60 to 1.03	0.080
Time of exposure (daily)						
No or sporadic exposure	1^e^			1^e^		
< 1–3 hours	1.14	0.84 to 1.54	0.403	0.87	0.63 to 1.22	0.427
More than 3 hours	0.56	0.34 to 0.93	0.020	0.68	0.45 to 1.04	0.078
***Exposure at indoor workplaces***^b^						
Close exposure (tobacco smoke reaches their workplace)	0.13^d^	0.09 to 0.19	*< 0.001*	0.15^d^	0.10 to 0.25	< 0.001
Time of exposure (daily)						
No or sporadic exposure	1^e^			1^e^		
< 1–3 hours	0.10	0.06 to 0.17	*< 0.001*	0.11	0.05 to 0.24	< 0.001
More than 3 hours	0.14	0.09 to 0.22	*< 0.001*	0.10	0.06 to 0.18	< 0.001
***Exposure in bars and restaurants***^c^						
Environmental tobacco smoke in bars						
None	1^e^			1^e^		
Low level	0.60	0.38 to 0.95	0.029	0.49	0.24 to 0.99	0.047
Fairly high level	0.29	0.18 to 0.46	*< 0.001*	0.33	0.16 to 0.67	0.002
Very high level	0.18	0.11 to 0.29	*< 0.001*	0.12	0.05 to 0.27	< 0.001
Environmental tobacco smoke in restaurants						
None	1^e^			1^e^		
Low level	0.30	0.22 to 0.39	*< 0.001*	0.33	0.22 to 0.49	< 0.001
Fairly high level	0.21	0.15 to 0.30	*< 0.001*	0.30	0.17 to 0.51	< 0.001
Very high level	0.11	0.06 to 0.20	*< 0.001*	0.11	0.03 to 0.35	< 0.001

^a ^Households with more than one person

^b ^People who work in indoor places away from home

^c ^People who have visited such premises in the last month

^d ^Binary logistic regression. Odds ratios adjusted by sex, age, number of people in the household (only for exposure at home) and educational level. Reference categories: Nobody regularly smokes at home; No close exposure at workplace

^e ^Multinomial logistic regression. Odds ratios adjusted by the same variables as in the binary logistic regression models. First category = 1 is the reference category

## Discussion

The results of this study suggest that the "Tobacco control law" is having an important effect on the reduction of environmental exposure in the workplace and, to a lesser extent, in bars and restaurants.

Spain has been, along with Ireland, Norway, Malta, Italy and Sweden [9], one of the first European countries to introduce, at a national level, a law that bans smoking -with certain exceptions and variations- in enclosed public places. There are a large number of variations that are mainly focused on the possibility of setting up smoking areas. Except for these differences, the initial evaluations of the introduction of legislation that regulates smoking in these European countries describe an important effect in the control of the prevalence of passive exposure [10,11].

The most important effect revealed by this study is related to the indicators of exposure in enclosed spaces in the workplace, an environment in which the "Tobacco control law" totally bans smoking. Bearing in mind that the proportion of the total active population working in enclosed spaces outside the home is around 60%, and that one of every four workers was exposed to tobacco smoke for more than 3 hours, reduction of this exposure could have important preventive implications for the population [12]. Furthermore, a smoking ban in the workplace causes an absolute reduction of 4% in the prevalence of smoking among workers [13]. However, despite the important effect attributable to the law, 10.6% of smokers state that they still smoke in indoor workplaces, showing the need to establish mechanisms that guarantee compliance with the law.

Passive exposure has also been considerably reduced in bars and restaurants, although to a lesser extent than in other workplaces. This is consistent with the fact that the law establishes a partial ban on smoking in such premises. In addition, it should be taken into account that Spain was still in a period of adapting to the law. A period of eight months has been allowed for the installation of physically separated smoking areas, in those premises of more than 100 square metres that are permitted to have them. Nevertheless, considering the high percentage of people who have visited bars and restaurants in the last month and the high occupational exposures to second-hand smoke in workers of this sector, the law should be more restrictive with regard to tobacco consumption in these public spaces. In those countries where the limitation on smoking is more restrictive, a much greater effect has been seen than in our population [10,14,15].

The data for exposure at home are also consistent and similar to those of other studies, which have found few effects after legislation came into force [10]. The strategy for reducing passive exposure at home is much more complex, and it should be based on the development of comprehensive programmes of smoking control, with complementary control measures that are not currently considered [16].

Design limitations include that no geographical control area was available, as was the case, for example, of a study in Ireland [10], where the results were compared with those seen in the United Kingdom. In addition, although the sample size used guaranteed statistical power to undertake an overall analysis, it does not yet allow the identification of possible differences among population subgroups. Self-reported information from questionnaires is the method most commonly used for measuring passive exposure to tobacco smoke. Some authors have detected problems of sensitivity when comparing self-declared data with the study of biomarkers, which is probably due to the difficulties of identifying sources, duration and quantity of exposure. Yet other authors have observed a strong correlation [17].

The "Tobacco control law" has created a favourable climate for the prevention and control of smoking in Spain, and will probably have a greater impact when global strategies are developed, as it has been demonstrated that the more extensive and comprehensive the control strategies are, the more efficient the intervention is [18,19].

## Conclusion

This initial evaluation of the "Tobacco control law" emphasises the substantial effect it has had upon the control of exposure to environmental tobacco smoke in the workplace and, to a lesser extent, in bars and restaurants, while few changes are seen in exposure at home. These effects are consistent with the control measures introduced: total ban, partial ban or without controls, respectively. These encouraging results should contribute towards increasing compliance with the law, and to the development of new control mechanisms that will guarantee real smoke-free spaces.

## Competing interests

The author(s) declare that they have no competing interests.

## Authors' contributions

IG had the original idea and was responsible for the design of the study, statistical analyses and preparation of the manuscript. NM contributed to the design of the study, statistical analyses and preparation of the manuscript. CE contributed to the preparation of the manuscript. LD contributed to the analysis and interpretation of the data, and also revised the manuscript. LV oversaw the study and revised the manuscript. BZ, AG and HO contributed to the discussion of the results and revised the manuscript. All authors read and approved the final manuscript.

## Pre-publication history

The pre-publication history for this paper can be accessed here:


